# Improving epidemiologic data analyses through multivariate regression modelling

**DOI:** 10.1186/1742-7622-10-4

**Published:** 2013-05-17

**Authors:** Fraser I Lewis, Michael P Ward

**Affiliations:** 1 Section of Epidemiology, VetSuisse Faculty, University of Zürich, Winterthurerstrasse 270, Zürich, CH 8057, Switzerland; 2 Faculty of Veterinary Science, University of Sydney, Camden, NSW 2570, Australia

## Abstract

Regression modelling is one of the most widely utilized approaches in epidemiological analyses. It provides a method of identifying statistical associations, from which potential causal associations relevant to disease control may then be investigated. Multivariable regression – a single dependent variable (outcome, usually disease) with multiple independent variables (predictors) – has long been the standard model. Generalizing multivariable regression to multivariate regression – all variables potentially statistically dependent – offers a far richer modelling framework. Through a series of simple illustrative examples we compare and contrast these approaches. The technical methodology used to implement multivariate regression is well established – Bayesian network structure discovery – and while a relative newcomer to the epidemiological literature has a long history in computing science. Applications of multivariate analysis in epidemiological studies can provide a greater understanding of disease processes at the population level, leading to the design of better disease control and prevention programs.

## Introduction

Multivariable regression modelling in which multiple independent variables are regressed on a single dependent variable is a technique familiar to any epidemiologist. This analytical approach is a regular feature in the epidemiological literature, and is without doubt a useful tool. By extending this approach to an analogous multivariate regression model, in which all variables are simultaneously considered, substantially enhanced insight into the disease system under study may be gained. At worst, both multivariable and multivariate approaches will give identical results —as they must, because to determine the best possible multivariate model of study data, all possible multivariable models must also be considered, as the latter are simply special cases of the former.

Gaining additional insights into a disease system by simply switching to a more general data analytic technique is clearly very attractive, in particular when the theoretical foundations for the more general approach are long established. The modelling methodology we consider here is referred to as Bayesian network analysis (as defined in [1,2]). This is a form of graphical modelling [3,4], but whose focus is on structure discovery: determining an optimal statistical model, i.e. graphical structure, directly from observed data. Whilst relatively uncommon in the epidemiological literature, Bayesian network analyses are increasingly finding application in areas of biology, medicine and ecology (e.g. [5-12]) and Bayesian network modelling itself has a vast technical literature (as is easily seen by using the search term “Bayesian network” in any bibliographic database, e.g. pubmed, web of knowledge).

Identifying causal relationships is the objective of many epidemiological analyses involving regression modelling. Empirical analyses of epidemiological data can demonstrate statistical dependency between variables, and as we later demonstrate Bayesian network analysis is ideally suited to such a task. While the identification of statistical dependency is often a natural step towards postulating causal mechanisms, it is, however, vastly more ambitious to further assert that any given dependency exists within a particular causal web. Expert knowledge and biological understanding is clearly essential, since this is more than a statistical data analysis exercise. To avoid any unnecessary confusion, all analyses and discussion here pertain only to models of statistical association —it is a common misinterpretation to assume that arcs in a Bayesian network model denote causality, they denote only statistical dependency.

Our objective here is to demonstrate the potential utility of Bayesian network structure discovery to epidemiologists. We consider specifically additive Bayesian networks, which are Bayesian network models parameterized in an analogous fashion to generalised linear models. The classical formulation of Bayesian networks for binary or multinomial variables uses a mathematically elegant contingency table parameterisation [1,2]. For epidemiological analyses such a parameterisation is both unusual and rather opaque, and is likely vastly over parameterized compared to the familiar additive formulation used in generalised linear models (as discussed in [13]).

In the following sections we first briefly review the motivation and experimental origins of regression modelling in scientific studies. Graphical regression is then introduced, followed by a series of simple empirical examples which compare and contrast multivariable and multivariate regression. We then discuss the epidemiological implications of these results and the limitations of the approach.

## Regression modelling concepts - a brief review

In classical experimental trial scenarios (e.g. [14], such as factorial or Latin square designs), the investigator is able to fix at predetermined values all of the variables of interest in the experiment. These are the independent variables in a multivariable regression model. The research question being asked here is how the measurement variable – the outcome or response variable – changes across the various different patterns of values chosen for the independent variables. This is the historical foundation of regression modelling. The ability to fix all variables of interest to predetermined values is crucial and underlies the experimental study design, because it enables unambiguous estimation of all key covariate effects on the response variable.

The classical experimental design scenario contrasts sharply with what is feasible and practical in many epidemiological studies, either in humans or other animals. Considering zoonotic pathogens for example, animal husbandry, livestock production and farm environment characteristics are by their nature highly inter-dependent. Thus, it is generally impossible to separate out the “true” effects of individual covariates on the response variable (e.g. the design matrix is not orthogonal, see [15]) because the estimated effect of any covariate will now generally also depend on what other covariates are also included in the model (including the case in which all variables are included). Moreover, determining the most appropriate covariates for inclusion in the model is considerably more difficult when dependencies exist between study variables, as in the case in which confounding variables are present. The Yule-Simpson paradox [16-18]: that an apparent relationship between variables (e.g. a disease and a putative risk factor) may disappear or even be reversed when other variables are taken into account, is particularly troublesome here. Similarly, the closely related difficulties of negative (or positive) confounding.

In multivariable regression, relationships between the “independent” variables in a study do not feature explicitly in the modelling process. This seems entirely reasonable in the classical designed experiment scenario. In regression analyses of epidemiologic data where many inter-dependencies between study variables may be present, explicitly modelling all relationships between all variables is intuitively far more reasonable (as demonstrated in our later examples). Common multivariable model selection approaches, such as stepwise searches, may be sufficient to implicitly account for such inter-dependencies, and thus identify an optimal set of predictors for the outcome (disease) variable. But a considerable difficulty here is how to justify that the modelling results obtained are as optimal as is practicable for a given study. The standard way to address such issues in statistical modelling is to compare a simpler model with a more general model. If the goodness of fit of the simpler model is no worse than the more general model then the former is chosen as the preferred model. This is the concept of parsimony —it is more desirable to explain a phenomenon, e.g. disease occurrence, with a simpler than a complex model. In our current context “more general” also refers to expanding the scope of the modelling framework to explicitly include all relationships between all variables, i.e. a multivariate rather than multivariable regression model. The Bayesian network literature has long provided all the necessary theory and algorithms (e.g. [1,2,19,20]) to implement such regression modelling. Historically, the main practical difficulty in the application of this approach has been a lack of suitable computing resources and relevant accessible software.

## Regression modelling in epidemiology

In typical regression analyses found in the epidemiological literature (e.g. [21,22]) the use of a hypothesis testing (P-values) framework is still far more common than Bayesian inference. There is a considerable body of evidence which strongly argues against the use of hypothesis testing and P-values for model comparison and selection. Information theoretic and Bayesian approaches are argued to be preferable on both conceptual and performance grounds [23-26]. When the primary objective is to identify optimal parsimonious models, i.e. structure discovery, then, in purely practical terms, using a Bayesian or non-Bayesian paradigm is largely irrelevant as in such analyses the use of uninformative or diffuse priors is the standard practice in structure discovery (e.g. see [1,2,19,20]). Hence, the actual parameter estimates in any given model will be almost identical to the maximum likelihood analogue. However, the very considerable advantage of adopting a Bayesian paradigm is that we can then directly utilize established model selection and comparison techniques from the Bayesian networks literature [2,19,20].

## Empirical examples: multivariable versus multivariate

We first briefly describe a graphical statistical model, recall that additive Bayesian network structure discovery is concerned entirely with graphical models, and its conceptual differences from classical regression. We then present three separate illustrative analyses using risk factor case study data (unpublished veterinary data with variable names anonymized to maintain confidentiality) comprising of 400 observations across 17 variables, where each variable is a measurement or attribute from an individual subject (animal) and each subject only appears once in the data. There are five binary variables and 12 continuous variables. Note that for our current purposes background knowledge of the particular variables in the study is not relevant, as we are only interested in comparing and contrasting the statistical results obtained by applying two different techniques to identical data. This is an observational study and the investigator was not able to fix the values of any of these variables.

### Introducing graphical regression

**Figure 1:**
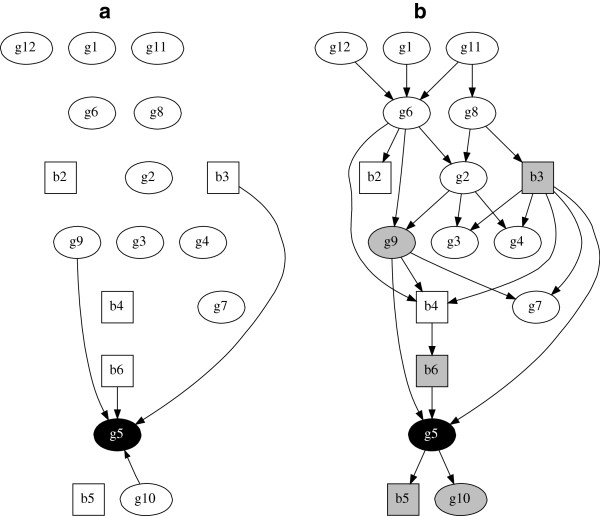
**Globally optimal multivariable regression model with g5 as the response variable and globally optimal multivariate regression model of all 17 variables.** (**a**) Globally optimal multivariable regression model with g5 as the response variable and covariates b3, b6, g9 and g10, log marginal likelihood = -8664.4; (**b**) Globally optimal multivariate regression model of all 17 variables, log marginal likelihood = -8311.6. Markov blanket for variable g5 are those variables in grey. Squares denote binary variables, ovals continuous.

In graphical statistical modelling there is no distinction made between covariates and a response variable. All are just “variables” as, formally speaking, a graphical statistical model is a representation of the joint probability distribution of all the random variables in the data. Figure 1(a) depicts a graphical model which is directly analogous to a classical multivariable regression model, as arcs terminate only at a single “response” variable (e.g. g5). But this model has a statistical interpretation which is radically different from that in classical regression, here: i) variables b3, b6, g9 and g10 are directly dependent with variable g5; ii) variables b3, b6, g9, g10 are all indirectly dependent with each other (via g5); and iii) all other variables are independent. In terms of i), direct dependence means there is an arc directly connecting these variables (in either direction). In terms of ii), in a graphical model all variables in the same component (collection of connected arcs —ignoring direction) are jointly statistically dependent. This means that knowing the value of one variable in this component can potentially provide information about the likely value of any other variable in this component (see [3,4]). If a variable has no arcs, either emanating from it or terminating at it, then it is statistically independent. In such a case knowing the value of any other variable in the model tells us nothing about the value of these variables.

All the graphical models we consider here are concerned only with statistical dependency, and arc direction in such models in no way implies any causal relationship. The direction of arcs is a result of the probability calculus required when dealing with models comprising of joint probabilities. In general, arc direction has no epidemiological interpretation because observed data alone cannot discriminate between arcs of opposite directions. This is simply a consequence of factorising joint probability distributions, and is typically referred to as likelihood equivalence (see p.1052 in [11] for a more general explanation, and [2] for technical details). A potential practical complication of likelihood equivalence is when searching for an optimal graphical structure. Standard search approaches in the literature, such as Heckerman’s heuristic hill climber [2], and the exact order based search by Koivisto [20] (the latter is used in our later case studies), identify a single optimal (directed) graph. This is as opposed to all graphs within the same likelihood equivalence class, which is computationally intractable [2]. If the objective is to identify all statistical dependencies in study data then, as mentioned above, arc direction is not relevant and such difficulties can be ignored. This is not the case, however, when viewing the modelling results within a causal (or indeed a longitudinal) framework as the arc direction then has an obvious real interpretation. In causal analyses the use of *a priori* restrictions on arc directions to avoid contradicting known epidemiological fact is likely appropriate (although not without some conceptual challenges, see p212. in [2]). Causal analyses of data using graphical models represents a large, and somewhat distinct, literature from Bayesian networks, with [27] a standard text.

In summary, classical multivariable regression can easily be denoted by a graphical model, but where the interpretation of the model is different in that it is now a joint probability model, albeit of a very simple structure. The reason for considering such regression models within a graphical modelling framework is that the graphical structure can now easily be relaxed to allow dependencies (arcs) to be present between any variables, i.e. this framework allows us to directly compare results from applying multivariable regression and multivariate regression on the same data. This then gives us our main “result” of this paper —a demonstration of how using multivariate regression may enhance our understanding of a disease system.

### Case study results

We now present three analyses. In each we determine the globally optimal “multivariable” graphical regression model, and compare this with the globally optimal “multivariate” graphical regression model. The term “globally optimal” here refers to a model which has the best possible goodness of fit of all possible models, and is determined using an established exact (as opposed to heuristic) structural search algorithm [20]. The goodness of fit metric used here is the marginal likelihood [28], which is the standard metric in Bayesian model selection.

When comparing models in a Bayesian paradigm the objective is to infer which is the most plausible model given suitable observations. Borrowing notation and terminology from Mackay [28], the posterior probability of each Bayesian model, $P(H_{i}|D)$, can be written as $P(H_{i}|D)\propto P(D|H_{i})P(H_{i})$ where *D* denotes the observed data, e.g. a database of study records, and $H_{i}$ denotes hypothesis, in other words a model of the data, i.e. a chosen hypothesis about relationships in the data parameterized into a statistical model. The data dependent term, $P(D|H_{i})$ is called the *evidence*, and $P(H_{i})$ is a quantification of our subjective prior belief about the current hypothesis (i.e. model *i*) before any data has arrived. In the Bayesian networks literature is it usual for all models to be considered equally plausible prior to observing any data [2,19], in which case $P(H_{i})$ is just a fixed constant for all models (and thus can be ignored) and the evidence is proportional to the posterior probability for each model. The evidence, $P(D|H_{i})$, is also called the marginal likelihood and has been shown to have a number of theoretically desirable qualities, where the model with highest marginal likelihood is the preferred model. Model selection using the marginal likelihood has been shown to be equivalent to using Occam’s Razor (for more details see [28] p.422).

The model in Figure 1(a) is the best possible multivariable model for the data when we consider g5 as the response variable. That is, it is the best possible graph structure when an exact model search is used with the restriction that arcs are only allowed to terminate at variable g5. This search restriction ensures that the scope of our graphical model is limited only to a multivariable regression model. We now repeat an identical exact search but this time without the previous restriction on the location of arcs. This allows us to determine the best multivariate regression model of the data, that is, we consider all variables simultaneously. This model is given in Figure 1(b), and note that this is a directed acyclic graph (DAG), no cycles —feedback loops —exist which is a technical requirement of a graphical statistical model.

Before we compare the modelling results in Figure 1(a) and (b), it is worth emphasizing the key methodological point here: the only difference in the process which identified graph (a) as the best model of the observed data, and graph (b) as the best model of the observed data, is that in the former the scope of the model search was restricted to only consider graphs with arcs terminating at g5, i.e. a multivariable graphical regression model.

In a graphical model the standard way to interpret the results relative to a single variable is to compute its Markov blanket [29]. A Markov blanket (highlighted in grey in the figures) comprises the parents, children and children’s parents of the variable of interest (arcs go from parents to children [19]). To predict values for any variable in a DAG, then all we need to know are the variables in its Markov blanket, and all other variables in the graph can be discarded. Conversely, each variable in the Markov blanket is needed because each provides knowledge about the variable of interest.

In Figure 1(a) the multivariable model provides statistical evidence that variables b3, b6, g9 and g10 are directly dependent with g5. In Figure 1(b) the multivariate model provides evidence that additionally variable b5 is directly dependent with g5, and therefore obviously it is also in the Markov blanket of g5. This then suggests that b5 should be included along with these other variables for further investigation into their potential epidemiological significance with response g5. In summary, even although we only have a single response variable in this current analysis, using the more general multivariate model has provided a different set of most supported “predictor” variables.

**Figure 2:**
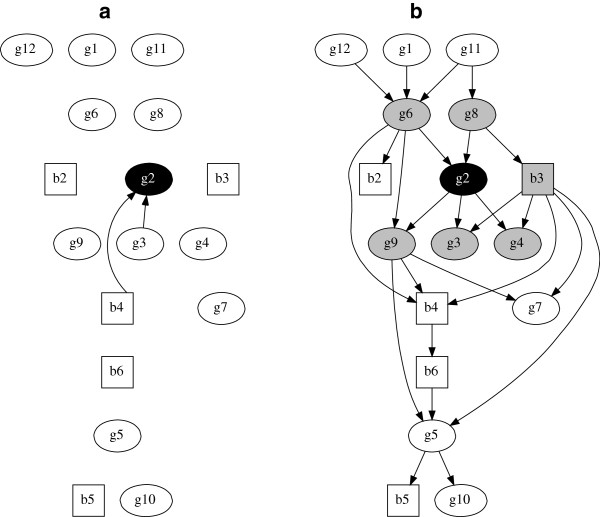
**Globally optimal multivariable regression model with g2 as the response variable and globally optimal multivariate regression model of all 17 variables.** (**a**) Globally optimal multivariable regression model with g2 as the response variable and covariates b4 and g3, blacklog marginal likelihood = -8530.0. (**b**) Globally optimal multivariate regression model of all 17 variables, blacklog marginal likelihood = -8311.6. Markov blanket for variable g2 are those variables in grey. Squares denote binary variables, ovals continuous.

**Figure 3:**
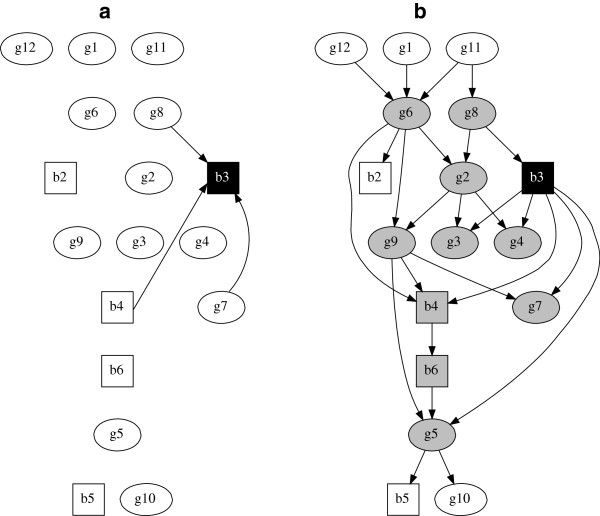
**Globally optimal multivariable regression model with b3 as the response variable and globally optimal multivariate regression model of all 17 variables.** (**a**) Globally optimal multivariable regression model with binary variable b3 as the response and covariates b4, g7 and g8, blacklog marginal likelihood = -8670.9. This is a generalised linear model with logit link function. (**b**) Globally optimal multivariate regression model of all 17 variables, blacklog marginal likelihood = -8311.6. Markov blanket for variable b3 are those variables in grey. Squares denote binary variables, ovals continuous.

We now consider two further examples which are analogous to the g5 example but which now treat variable g2 (Figure 2) and then variable b3 (Figure 3) as the response variables in multivariable analyses. The globally optimal multivariate model is obviously unchanged from Figure 1(b) since in such a model all variables are considered jointly, but the Markov blankets for g2 (Figure 2b) and b3 (Figure 3b) are now highlighted.

It is apparent that in both Figure 2 and Figure 3 the results obtained using multivariable regression are quite different from those obtained using multivariate regression. The number of direct arcs connected to (or from) the response variable have increased, from two to five in Figure 2 and from three to six in Figure 3. As the multivariate model permits arcs both to and from the response variable this is perhaps no surprise, although there is no reason that this need always be the case. What may be rather more surprising is that arcs identified in the multivariable model may not be identified in the multivariate model. For example in Figure 2(a) there is an arc from b4 to g2, but in Figure 2(b) b4 is not directly connected to g2 – moreover, it is not even in g2’s Markov blanket. The multivariable model suggests b4 is worthy of further investigation. In contrast, the full multivariate model suggests that in fact b4 is only indirectly related with g2, and this indirect dependence is also remote in the graph, i.e. outside the Markov blanket. In other words there is little statistical evidence to support epidemiological investigation of b4. This result cannot be dismissed by arguing that the multivariable model is somehow more parsimonious, because the same model selection metric is used in all model comparisons. blackThere is a very large difference (>100) in (log) marginal likelihood values between the multivariable and multivariate models (see figure legends). A guide to the relative size and interpretation of differences in (log) marginal likelihoods can be found in Table 2.1 page 27 in [30], and using the terminology there, a difference of greater than 10 is considered very strong evidence in favour of the model with greater (log) marginal likelihood. In summary, therefore, the data supports that the multivariate models are simply a better fit to the data in our examples.

Our final example is shown in Figure 3. The key difference between the results here is that the multivariable model implies that there are three variables worthy of further investigation. In contrast, the multivariate model has ten variables in the Markov blanket for b3, six of which are directly connected with b3.

To complete our case study analyses, and further emphasize that our proposed multivariate regression approach is simply a generalization of usual multivariable regression, it is readily possible to compute odds ratios and mean effects of the parameters (arcs) in our graphical model. For example, the marginal (posterior) log odds ratio for the arc from g5 to b5 (see panel b of any of the figures) has a 95% confidence (or credible) interval of (0.20,0.65). This is a log odds ratio as we have a logistic regression between b5 and g5 in this part of the graph. Similarly, the marginal mean (posterior) effect for the arc from b3 to g4 has a 95% confidence interval of (0.18,0.54), and for the arc from g2 to g9 the corresponding interval is (0.08,0.27). The latter two intervals are for the mean effect rather than log odds as these are Gaussian regressions. It is straightforward to compute such parameter coefficients for any node in the model, and note also that each of these 95% confidence intervals does not include zero. These would therefore typically be considered as having a strong degree of statistical support, and each is connected to the “response” node in each of our three multivariate examples.

Tables giving medians and marginal 95% posterior confidence intervals for every parameter in each of the three multivariable models (Figures 1a, 2a and 3a) and in the full multivariate model (Figures 1b/2b/3b) can be found in the Additional file 1: Appendix. A key point of note here is that nodes with the same parents have identical parameter estimates in each model (e.g. compare variables *g*1, *g*11 and *g*12 between the multivariable and multivariate models) as they should. The multivariate model is simply a collection of multivariable models and so the parameter estimates will be the same given the same parents. The difference is that the former is more flexible and allows any node to have parents, unlike in a GLM type model. Generally speaking —and as we have seen in our case study examples —this means that the parents, and therefore parameter estimates, may be different for at least some variables (nodes) in the data (e.g. compare the parameter estimates for node *g*5 between Figure 1a and 1b).

## Epidemiological implications

When the analytical task is to identify statistical dependencies with one (or more) response variables, then both theoretically and as demonstrated in the above empirical examples, the more general additive Bayesian network structure discovery approach appears clearly preferable. In particular, the multivariable approach is just a special case of the multivariate approach, i.e. there is nothing preventing the more general structural search (Figures 1b, 2b, 3b) from identifying the same globally optimal model as in the restricted structural search (Figures 1a, 2a, 3a). Hence, there is nothing to lose, at least in statistical terms, by adopting the more general approach. Moreover, the far simpler multivariable approach may identify covariates which are not supported by the multivariate model, e.g. the second case study example. A possible explanation for such contradictions is the Yule-Simpson paradox, in that we are trying to describe observations from a complex disease system of inter-dependent variables through a multivariable model, which may just be too simplistic for this particular application.

By using a multivariate regression approach the trade-off being made with classical multivariable regression is that the former may provide potentially more information about the disease system under study, in terms of identifying statistical dependencies. This may lead to new and novel findings. But equally, some of the newly identified statistical dependencies may also be readily discarded as potential causal associations, when viewed through the prism of an epidemiologist’s expert knowledge of the biology of the disease(s) of interest. A brief contrast may be made here with historical approaches such as path analyses [31], which were applied reasonably commonly during the 1970s to address a range of chronic and environmental diseases [32-34], and this approach still appears occasionally in the epidemiological literature. The key distinction between path analyses and additive Bayesian network structure discovery is that the former is explicitly causal, where some or all, of the graph structure is determined apriori via expert opinion. The latter asserts only the presence of statistical dependency, and while it can include prior expert opinion into the structural search (it is a Bayesian approach after all) the default usage is to allow the data itself to identify an optimal graph structure. The advantage of allowing the study data itself to identify an optimal graph is that this may include arcs which an expert may not, and may not include arcs which an expert would assert must be present in the given disease system. The epidemiological challenge is then to explain such discrepancies which may result in gaining new insight into the disease system.

## Software for multivariate regression

Reliable, easy to use software is essential for facilitating the uptake of any new data analytic technique into the epidemiological community. In order to apply additive Bayesian network structure discovery to study data appropriate software is required. In theory, Bayesian network analyses could be performed within a number of widely used Bayesian software programs, such as WinBUGS/OpenBUGS [35] or JAGS [36]. In practice, however, other approaches are necessary because the central task in Bayesian network analyses is structure learning which involves fitting and comparing a great many different multivariate models. In programs such as OpenBUGS and JAGS it is simply computationally impractical to fit every model via Markov chain Monte Carlo simulation, in addition to the difficulty in reliably estimating the marginal likelihood for each model. Instead, programs which employ analytical approximations, i.e. Laplace approximations [37,38], are preferable and indeed arguably necessary. The particular software we used in the examples is the abn library for R [39], which has been developed by the authors for performing additive Bayesian network structure discovery with epidemiological data and is available from CRAN (http://cran.r-project.org/web/packages/abn/index.html). This library has been extensively tested and validated against other established Bayesian modelling software such as INLA [40] (and also JAGS). The abn library also includes wrappers to allow INLA to be used for all numerical estimation. Case studies which demonstrate how to implement analyses similar to those presented, along with detailed numerical validation studies are available from http://www.r-bayesian-networks.org. A range of other R libraries for fitting Bayesian networks and other forms of graphical regression can be found at http://cran.r-project.org/web/views/gR.html. While these software libraries are all for use with R it is also possible for R to be accessed from within other popular statistical software such as SAS (via IML Studio).

## Limitations

### Computational feasibility

The main limitation when applying additive Bayesian network structure discovery to epidemiological data is computational feasibility. The number of variables which can be included in a Bayesian networks analysis is limited. As a guide, this might be less than about 25 variables for exact structural search techniques and perhaps up to 40 for heuristic approaches (e.g. see [11]). Inclusion of more variables is possible with access to specialist computing facilities and expertise. This means that including additional variables, such as interaction terms, which can be done easily enough just as in standard regression modelling, requires careful consideration. Each term adds to the number of variables in the model, and therefore adds considerably to the computational time required to perform structural searches. There are a number of ad-hoc ways to address the computational demands. For example by splitting variables into smaller thematic groups for analyses. This may then suggest that some variables can be dropped, reducing the computationally burden to a more manageable level. For larger problems (more variables), model averaging using order-based Markov chain Monte Carlo is an option [19] as it can cope with many more variables (e.g. >100). Such averaging approaches randomly sample from the posterior landscape of possible graphs (strictly speaking, node orderings), with better fitting graphs being sampled more often than poorer fitting graphs, and during this sampling (i.e. jumping from model to model) it is possible to estimate the relative support for each arc (or groups of arcs) in terms posterior probabilities. This is an approach used in bioinformatics for sequence analyses (e.g. [10]) but does have some important caveats, such as producing results which will be biased relative to direct (non-order based) model selection.

Access to scientific computing facilities, while not essential, is highly beneficial. For larger problems (>25 variables), heuristic search approaches are required which are demanding as they must be run many times to ensure reliable results. An additional severe computational drain is addressing over-fitting, which is an ever present problem in model selection [41], irrespective of whether using exact or heuristic searches. Good practice in structure discovery is to either utilize some form of model averaging, for example using majority consensus graphs as the optimal model [9,42], or else using parametric bootstrapping approaches [43] applied to the globally best graph [11]. The majority consensus approach is similar to that used in phylogenetics with tree structures, except here a majority consensus graph is created from all arcs which appeared in at least a majority of heuristic search results. This provides an alternative way to estimate relative support for individual arcs other than by Markov chain Monte Carlo, which can be highly problematic when dealing with graph structures (see [19]). A single exact search for a model comprising of 20 variables may take 24 hours to complete on a modern desktop, and this may need to be repeated many (hundreds) times during model averaging or bootstrapping to ensure robust results. Code for addressing over-fitting using parametric bootstrapping and also parallelization across a cluster computer can be found at http://www.r-bayesian-networks.org.

## Future potential: missing values

Missing observations are a common feature of field studies and epidemiological data. In standard regression modelling, observations with missing values are usually dropped from analyses (as it is essential to maintain identical observations when comparing different models). In graphical regression modelling this is also the easiest course of action. There are, however, a number of established algorithms for fitting graphical models in the presence of missing values due to the joint probabilistic nature of these models. Rather than “fill-in” such values using traditional approaches such as multiple-inputation, a graphical model can be used to either marginalize out missing entries in the data [44] or predict their most likely values using the graphical structure itself via the propagation of probabilities across the graph (methods of propagation form a considerable part of the graphical modelling literature, see [4,45,46]). These are elegant conceptual solutions, although they do still assume that values are missing at random, but such approaches are numerically highly complex. It is unclear whether these would be feasible in the context of structure discovery, as when there are missing values in the data the graph can no longer be split into conditionally independent computational units (i.e. each node and its parents - for estimating the marginal likelihood, see [2]). This is a very considerable complication, both in terms of implementation and computing time. Approaches have been developed for structure discovery in the presence of missing values, such as Structural-EM [47], although implemented in models with a simpler parameterisation than those presented here. The implementation of such approaches is an area of future work, but further highlights the considerable existing theory and potential of graphical regression and structure discovery approaches in analyses of epidemiological data.

## Conclusion

The wide utilization of regression modelling in epidemiological analyses means that outputs from such analyses have a ready application in disease control and prevention programs. Up until recently, such applications have been constrained by the use of multivariable regression. Extending multivariable regression to full multivariate regression —utilizing additive Bayesian network structure discovery —offers the epidemiologist potentially far greater insight into the complex inter-relationships between variables within a disease system. The main constraint in the use of this methodology is its considerable computational demands, but given the ever increasing availability of cheap computing power this technique is increasingly feasible for use in a wide range of studies.

## Competing interests

Both authors have no financial or non-financial competing interests to declare.

## Authors’ contributions

FIL wrote the manuscript and performed the statistical modelling, MPW co-wrote and assisted with the manuscript. Both authors read and approved the final manuscript.

## Supplementary Material

Additional file 1: Appendix Supplementary tables of parameter estimates. Click here for file
