# Supplementary material for: Improving epidemiologic data analyses through multivariate regression modelling
Source: Emerg Themes Epidemiol. 2013 May 17;10:4. doi: 10.1186/1742-7622-10-4 (PMC3691873; doi:10.1186/1742-7622-10-4)
Supplement: Additional file 1: Appendix — Supplementary tables of parameter estimates. [file 1742-7622-10-4-S1.pdf]

Appendix

Supplementary tables of parameter estimates

The observed data (continuous variables only) were standarised to mean zero and standard deviation of unity prior to any model fitting. This is common practice in Bayesian inference and model selection as it enables easier and more reliable numerical estimation of the parameters in the model while having no effect on the level of statistical support for model features (c.f. in a classical - non-Bayesian - regression analysis the p-values obtained are unaffected by this standardisation of the data).

Parameters in Figure 1 (a): Response $g5$

Node	Arc	Median & 95% CI	Node	Arc	Median & 95% CI
$b2$	Intercept	-1.27 (-1.50, -1.05)	$g5$	$g10 \rightarrow g5$	-0.30 (-0.38, -0.21)
$b3$	Intercept	-0.91 (-1.12, -0.71)	$g5$	precision	1.26 (1.10, 1.44)
$b4$	Intercept	-2.61 (-3.00, -2.25)	$g6$	Intercept	0.00 (-0.09, 0.09)
$b5$	Intercept	-0.13 (-0.32, 0.04)	$g6$	precision	1.00 (0.87, 1.14)
$b6$	Intercept	-1.91 (-2.20, -1.64)	$g7$	Intercept	0.00 (-0.09, 0.09)
$g1$	Intercept	0.00 (-0.09, 0.09)	$g7$	precision	1.00 (0.87, 1.14)
$g1$	precision	1.00 (0.87, 1.14)	$g8$	Intercept	0.00 (-0.09, 0.09)
$g2$	Intercept	0.00 (-0.09, 0.09)	$g8$	precision	1.00 (0.87, 1.14)
$g2$	precision	1.00 (0.87, 1.14)	$g9$	Intercept	0.00 (-0.09, 0.09)
$g3$	Intercept	0.00 (-0.09, 0.09)	$g9$	precision	1.00 (0.87, 1.14)
$g3$	precision	1.00 (0.87, 1.14)	$g10$	Intercept	0.00 (-0.09, 0.09)
$g4$	Intercept	0.00 (-0.09, 0.09)	$g10$	precision	1.00 (0.87, 1.14)
$g4$	precision	1.00 (0.87, 1.14)	$g11$	Intercept	0.00 (-0.09, 0.09)
$g5$	Intercept	-0.16 (-0.27, -0.06)	$g11$	precision	1.00 (0.87, 1.14)
$g5$	$b3 \rightarrow g5$	0.34 (0.15, 0.52)	$g12$	Intercept	0.00 (-0.09, 0.09)
$g5$	$b6 \rightarrow g5$	0.52 (0.27, 0.77)	$g12$	precision	1.00 (0.87, 1.14)
$g5$	$g9 \rightarrow g5$	0.21 (0.12, 0.29)			

Parameters in Figure 2 (a): Response $g2$

Node	Arc	Median & 95% CI	Node	Arc	Median & 95% CI
$b2$	Intercept	-1.27 (-1.50, -1.05)	$g5$	precision	1.00 (0.87, 1.14)
$b3$	Intercept	-0.91 (-1.12, -0.71)	$g6$	Intercept	0.00 (-0.09, 0.09)
$b4$	Intercept	-2.61 (-3.00, -2.25)	$g6$	precision	1.00 (0.87, 1.14)
$b5$	Intercept	-0.13 (-0.32, 0.04)	$g7$	Intercept	0.00 (-0.09, 0.09)
$b6$	Intercept	-1.91 (-2.20, -1.64)	$g7$	precision	1.00 (0.87, 1.14)
$g1$	Intercept	0.00 (-0.09, 0.09)	$g8$	Intercept	0.00 (-0.09, 0.09)
$g1$	precision	1.00 (0.87, 1.14)	$g8$	precision	1.00 (0.87, 1.14)
$g2$	Intercept	0.03 (-0.02, 0.10)	$g9$	Intercept	0.00 (-0.09, 0.09)
$g2$	$b4 \rightarrow g2$	-0.52 (-0.77, -0.27)	$g9$	precision	1.00 (0.87, 1.14)
$g2$	$g3 \rightarrow g2$	0.73 (0.66, 0.79)	$g10$	Intercept	0.00 (-0.09, 0.09)
$g2$	precision	2.23 (1.95, 2.54)	$g10$	precision	1.00 (0.87, 1.14)
$g3$	Intercept	0.00 (-0.09, 0.09)	$g11$	Intercept	0.00 (-0.09, 0.09)
$g3$	precision	1.00 (0.87, 1.14)	$g11$	precision	1.00 (0.87, 1.14)
$g4$	Intercept	0.00 (-0.09, 0.09)	$g12$	Intercept	0.00 (-0.09, 0.09)
$g4$	precision	1.00 (0.87, 1.14)	$g12$	precision	1.00 (0.87, 1.14)
$g5$	Intercept	0.00 (-0.09, 0.09)			

Parameters in Figure 3 (a): Response $b3$

Node	Arc	Median & 95% CI	Node	Arc	Median & 95% CI
$b2$	Intercept	-1.27 (-1.50, -1.05)	$g5$	Intercept	0.00 (-0.09, 0.09)
$b3$	Intercept	-1.18 (-1.43, -0.94)	$g5$	precision	1.00 (0.87, 1.14)
$b3$	$b4 \rightarrow b3$	2.22 (1.35, 3.22)	$g6$	Intercept	0.00 (-0.09, 0.09)
$b3$	$g7 \rightarrow b3$	0.61 (0.35, 0.89)	$g6$	precision	1.00 (0.87, 1.14)
$b3$	$g8 \rightarrow b3$	-0.48 (-0.75,-0.23)	$g7$	Intercept	0.00 (-0.09, 0.09)
$b4$	Intercept	-2.61 (-3.00, -2.25)	$g7$	precision	1.00 (0.87, 1.14)
$b5$	Intercept	-0.13 (-0.32, 0.04)	$g8$	Intercept	0.00 (-0.09, 0.09)
$b6$	Intercept	-1.91 (-2.20, -1.64)	$g8$	precision	1.00 (0.87, 1.14)
$g1$	Intercept	0.00 (-0.09, 0.09)	$g9$	Intercept	0.00 (-0.09, 0.09)
$g1$	precision	1.00 (0.87, 1.14)	$g9$	precision	1.00 (0.87, 1.14)
$g2$	Intercept	0.00 (-0.09, 0.09)	$g10$	Intercept	0.00 (-0.09, 0.09)
$g2$	precision	1.00 (0.87, 1.14)	$g10$	precision	1.00 (0.87, 1.14)
$g3$	Intercept	0.00 (-0.09, 0.09)	$g11$	Intercept	0.00 (-0.09, 0.09)
$g3$	precision	1.00 (0.87, 1.14)	$g11$	precision	1.00 (0.87, 1.14)
$g4$	Intercept	0.00 (-0.09, 0.09)	$g12$	Intercept	0.00 (-0.09, 0.09)
$g4$	precision	1.00 (0.87, 1.14)	$g12$	precision	1.00 (0.87, 1.14)

Parameters in Figure 1/2/3 (b): Full BN model

Node	Arc	Median & 95% CI	Node	Arc	Median & 95% CI
$b2$	Intercept	-1.37 (-1.63, -1.13)	$g5$	$b3 \to g5$	0.38 (0.19, 0.58)
$b2$	$g6 \to b2$	-0.60 (-0.89, -0.33)	$g5$	$b6 \to g5$	0.50 (0.24, 0.76)
$b3$	Intercept	-0.99 (-1.22, -0.77)	$g5$	$g9 \to g5$	0.25 (0.16, 0.34)
$b3$	$g8 \to b3$	-0.61 (-0.87, -0.36)	$g5$	precision	1.14 (0.99, 1.30)
$b4$	Intercept	-5.91 (-7.55, -4.64)	$g6$	Intercept	0.00 (-0.08, 0.08)
$b4$	$b3 \to b4$	2.74 (1.78, 3.84)	$g6$	$g1 \to g6$	0.24 (0.16, 0.32)
$b4$	$g6 \to b4$	-2.11 (-3.09, -1.29)	$g6$	$g11 \to g6$	-0.41 (-0.49, -0.33)
$b4$	$g9 \to b4$	-1.46 (-2.50, -0.59)	$g6$	$g12 \to g6$	0.15 (0.07, 0.24)
$b5$	Intercept	-0.13 (-0.32, 0.05)	$g6$	precision	1.34 (1.17, 1.53)
$b5$	$g5 \to b5$	0.42 (0.20, 0.65)	$g7$	Intercept	-0.15 (-0.25, -0.04)
$b6$	Intercept	-1.83 (-2.12, -1.55)	$g7$	$b3 \to g7$	0.52 (0.32, 0.72)
$b6$	$b4 \to b6$	-22.5 (-68.55,-2.85)	$g7$	$g9 \to g7$	0.17 (0.08, 0.26)
$g1$	Intercept	0.00 (-0.09, 0.09)	$g7$	precision	1.09 (0.95, 1.24)
$g1$	precision	1.00 (0.87, 1.14)	$g8$	Intercept	0.00 (-0.09, 0.09)
$g2$	Intercept	0.00 (-0.08, 0.08)	$g8$	$g11 \to g8$	-0.26 (-0.35, -0.17)
$g2$	$g6 \to g2$	0.28 (0.19, 0.37)	$g8$	precision	1.07 (0.93, 1.22)
$g2$	$g8 \to g2$	-0.23 (-0.32, -0.14)	$g9$	Intercept	0.00 (-0.08, 0.08)
$g2$	precision	1.13 (0.98, 1.28)	$g9$	$g2 \to g9$	0.17 (0.08, 0.27)
$g3$	Intercept	-0.08 (-0.15, 0.00)	$g9$	$g6 \to g9$	0.20 (0.11, 0.29)
$g3$	$b3 \to g3$	0.28 (0.14, 0.42)	$g9$	precision	1.09 (0.95, 1.25)
$g3$	$g2 \to g3$	0.72 (0.65, 0.78)	$g10$	Intercept	0.00 (-0.08, 0.08)
$g3$	precision	2.22 (1.94, 2.53)	$g10$	$g5 \to g10$	-0.34 (-0.42, -0.25)
$g4$	Intercept	-0.10 (-0.19, 0.00)	$g10$	precision	1.13 (0.98, 1.28)
$g4$	$b3 \to g4$	0.36 (0.18, 0.54)	$g11$	Intercept	0.00 (-0.09, 0.09)
$g4$	$g2 \to g4$	-0.50 (-0.58, -0.42)	$g11$	precision	1.00 (0.87, 1.14)
$g4$	precision	1.35 (1.18, 1.54)	$g12$	Intercept	0.00 (-0.09, 0.09)
$g5$	Intercept	-0.17 (-0.28, -0.06)	$g12$	precision	1.00 (0.87, 1.14)
